# Revisit the signatures of γδ T cells in hepatocellular carcinoma

**DOI:** 10.1002/ctm2.859

**Published:** 2022-05-11

**Authors:** Yanan Gao, Maojun You, Pengyuan Yang

**Affiliations:** ^1^ CAS Key Laboratory of Infection and Immunity CAS Center for Excellence in Biomacromolecules Institute of Biophysics University of Chinese Academy of Sciences, Chinese Academy of Sciences Beijing China

1

The liver is a critical hub of immunotolerance with preferentially enriched γδ T cells and other cells such as Kuffer cells, natural killer (NK) cells, conventional αβ T cells and B cells. But during the carcinogenesis of hepatocellular carcinoma (HCC), a progressive depletion of intrahepatic liver‐resident NK (LrNK) cells, cytolytic T cells and γδ T cells and an enrichment of regulatory T (Treg) cells and macrophages are critical involved correlating with tumour progression and prognoses.^1^ The immunological identification on the microenvironment of HCC and multiple sub‐class cells with the molecular classifications using single‐cell approaches opened the field to explore the cellular phenotypic diversities and functionalities. Nevertheless, the immune signatures of γδ T cells are not fully understood at the single‐cell level, particularly in the context of the HCC tumour microenvironment (TME). In this issue, He et al.^2^ reveal the immune landscape, functional states, metabolism, cytotoxicity and T cell receptor (TCR) profiles of HCC‐infiltrating γδ T cells using single‐cell RNA sequencing (scRNA‐seq), which may facilitate the development of γδ T cell‐based immunotherapy.

The γδ T cells are preferentially abundant in the liver, with 6.8%–34% γδ TCR expressed in the liver CD3^+^ T cells.^3^ As reported, γδ T cell infiltration is lower in HCC than in peri‐tumour tissues.^4^ However, the functional differences between γδ T cells in the HCC TME and normal liver tissue remain poorly understood. Although Vδ2 T cell therapy could significantly slow down the progression of liver cancer by its anti‐tumour immunity, another tumour‐infiltrated IL‐17‐producing γδ T cells exhibit as a tumour‐promoting function by inducing angiogenesis, making the role of tumour‐infiltrated‐γδ T cells more complicated.^5^, ^6^ Thus, further characterizing and distinguishing the fingerprints of tumour‐infiltrated γδ T cells will reveal their functional complexity, leading to more effective HCC immunotherapies.

In this study, He et al. explored the immune landscape and functional states of HCC‐infiltrating γδ T cells and performed scRNA‐seq on γδ T cells from healthy and HCC liver perfusates, peripheral blood of HCC patients, as well as ex vivo expanded Vγ9Vδ2 T cell from healthy donors. Clustering of scRNA‐seq profiles of γδ T cells revealed that the γδ T cells derived from HCC patients were dominantly enriched in cluster C4 and showed high level expressions of genes including *GADD45*γ, *LAG3*, *GNLY*, *GZMB* and *IFNG*, indicative of cytotoxic and exhausted phenotypes. Pathway enrichment analyses of the cluster C4 revealed that tumour‐infiltrating γδ T cells exhibited alterations in glutamine metabolism, apoptosis, TCR signaling, indicating the metabolic reprogramming and the loss of effector cell function of these γδ T cells.

TCR diversity seems crucial to overcome the natural genetic instability of cancers and their antigenic heterogeneity, which impacts the design of cellular therapies.^7^ To validate the finding of TCR signaling alteration, He et al. performed TCR clonality analyses, which indicated that the loss of TCR diversity was different in γδ T cells from HCC patients, with Vδ1 T cells enriched in HCC TME, while Vδ2 T cells enriched in peri‐tumour tissues and healthy liver tissues. Loss of TCR diversity in HCC tumour‐infiltrating γδ T cells may attribute to the limited T cell proliferation ability and enhanced apoptosis in the TME. Therefore, He et al. analyzed the cell cycle phases and found that nearly 60% of γδ T cells in cluster C4 were in the G2/M phase, indicative of the cell cycle arrest of γδ T cells in the TME. In contrast, other γδ T cells were mainly enriched in phase G1. Furthermore, He et al. evaluated the expression of cytotoxic and inhibitory genes at the transcriptional and protein levels. Interestingly, γδ T cells from HCC patients particularly up‐regulated LAG3, but not other checkpoint molecules, suggesting a LAG3‐mediated exhaustion phenotype of HCC tumour‐infiltrating γδ T cells.

Cellular metabolism has been recognized as a critical determinant of the viability and function of immune cells. Evaluating the immune metabolism can uncover metabolic vulnerabilities and therapeutic windows upon which to intervene for enhanced immunotherapy.^8^ He et al. further focused on alterations in the major metabolic pathways of γδ T cells in the HCC TME. Up‐regulated genes including *SLC1A5*, *OAT* and *GLS* in HCC tumour‐infiltrating γδ T cells may associate with up‐regulated glutamine metabolic reprogramming. Besides, in the in vitro glutamine restriction and glutamine inhibitory experiments, γδ T cells displayed enhanced expression of LAG3 but decreased the expression of IFNγ and TNFα. Cytokine/chemokine assays revealed that proinflammatory cytokines, such as IFNγ, MIP1a, MIP1b, IL8, IL13 and GM‐CSF, were increased at 24 h while decreased for prolonged exposure of γδ T cells to the glutamine‐deficient medium. These results indicate a LAG3‐dependent dysfunction for HCC‐infiltrating γδ T cells to secret proinflammatory cytokines.

As ex vivo expanded Vδ2 T cells could rapidly migrate to and accumulate at tumour sites,^9^ He et al. also evaluated whether ex vivo expanded Vδ2 T cells could complement the loss of the TCR diversity and effector functionality of the HCC‐infiltrating γδ T cells. The scRNA‐seq profiling of the in vitro expanded Vδ2 γδ T cells derived from blood of healthy donors showed a high diversity of γδ TCR repertoire and effector functions which could complement the loss of the anti‐tumour immunity of HCC‐infiltrating γδ T cells, providing a potential strategy of the ex vivo expanded Vδ^2^ γδ T cells for immunotherapy, which needs to be further studied in the future. Besides, as numbers of Vδ^2^ γδ T cells were infiltrated in the peri‐tumour tissues, the reanalyzing these cells on the characters, functional states, cytotoxicity and TCR profiles, may provide a broad insight for immunotherapy.

Although immunotherapy has increasingly become one of the most promising treatment strategies for HCC, limited responses have been reported for clinical cases. γδ T cells display potent cytotoxicity, which usually does not depend on tumour‐associated antigens, towards a broad potential of hematological and solid tumours.^10^, ^11^ All in all, this study complements the understanding of HCC‐infiltrating γδ T cells to facilitate the development of γδ T cell‐based immunotherapy or checkpoint blockade combination immunotherapy. Further studies and analyses of γδ T cells in HCC are expected to assess signature and potential of the in vitro expanded Vδ2 γδ T cells recruited into tumour regions of liver cancer.

## CONFLICT OF INTEREST

The authors declare that there is no conflict of interest that could be perceived as prejudicing the impartiality of the research reported.
